# Lifestyle Therapy for the Treatment of Youth with Type 2 Diabetes

**DOI:** 10.1007/s11892-014-0568-z

**Published:** 2014-11-11

**Authors:** Jonathan McGavock, Allison Dart, Brandy Wicklow

**Affiliations:** 1 Department of Pediatrics and Child Health, Faculty of Medicine, Manitoba Institute of Child Health, University of Manitoba, 511 JBRC 715 McDermot ave., Winnipeg, MB R3E 3P4 Canada; 2 Diabetes Education and Resource Centre for Children and Adolescents, University of Manitoba, Winnipeg, Canada; 3 Section of Pediatric Nephrology, Department of Pediatrics, Faculty of Medicine, University of Manitoba, Winnipeg, Canada

**Keywords:** Exercise, Diet, Intensive lifestyle, Obesity, Diabetes, Adolescent, Mental health, Depression

## Abstract

Type 2 diabetes (T2D) in youth is a relatively novel condition facing paediatric health care providers. Few experimental trials exist to guide clinical management in this population. Supporting and prescribing modifiable lifestyle behaviours is cornerstone in the management of T2D in adults. Clinical trials in obese adolescents suggest that intensive lifestyle interventions that include both dietary changes and increased physical activity elicit clinically meaningful reductions in weight and improve cardiovascular risk profiles. Observational studies in youth with T2D suggest that better diet quality and increased physical activity are associated with better metabolic control; however, the limited experimental data available does not support these observations. Trials evaluating lifestyle monotherapy for the treatment of hyperglycaemia in youth with T2D do not exist, and the only study evaluating combined lifestyle and pharmacologic therapy did not show additional benefit over pharmacologic treatment with metformin alone. Physiological and psychosocial differences between youth and adults with T2D likely contribute to the differences in the effectiveness of lifestyle therapy for improving glycaemic control. The current review describes these topics in detail and provides recommendations for paediatric health care providers for the promotion of lifestyle therapy for the management of hyperglycaemia and cardiovascular risk factors for youth with T2DM.

## Introduction

Type 2 diabetes in children and adolescents is a novel chronic disease facing health care providers. As prevalence (0.18–1.2/1000) [1–5] and incidence rates (0.6–39/100,000 person years) [2–8] remain low, the majority of clinicians have had little opportunity to generate clinical experience managing hyperglycaemia and cardiovascular risk factors in youth with T2D. Similarly, very few therapeutic trials exist to guide clinical practice [9, 10••, 11]. Therefore, current management guidelines rely largely on data from adult studies and expert consensus [12, 13]. The purpose of this review is to summarize the evidence for the use of intensive lifestyle therapy for the management of youth with type 2 diabetes and provide recommendations for future research endeavours.

Youth with T2D are generally characterized by hyperglycaemia [14, 15], obesity [15–17], cardiovascular risk factor clustering [14, 17–19, 20•, 21] and adverse mental health outcomes [22–24]. These factors contribute to a rapid progression of diabetes-related complications [20•, 25], particularly renal disease [26, 27]. As adiposity is a primary driver for insulin resistance [28], hyperglycaemia [29] and a number of the comorbidities associated with type 2 diabetes (T2D) [30, 31], lifestyle management, defined herein as an increase in habitual physical activity and a change in dietary patterns that result in a daily caloric deficit and enhanced nutrient intake, is considered cornerstone in the management of T2D in youth [13, 32, 33]. The current review has three primary objectives related to the use of lifestyle management for the treatment of youth with T2D: (1) summarize the evidence for the efficacy of changes in lifestyle behaviours for improving body composition and cardiometabolic risk factors among obese adolescents; (2) discuss the roles of unique pathophysiology and poor mental health outcomes as determinants of the perceived failure of lifestyle therapy in youth with T2D and (3) highlight novel approaches for promoting lifestyle therapy in youth with T2D.

## Evidence for Lifestyle Management in Obese Adolescents

### Is Lifestyle Management Efficacious for Improving Body Composition in Obese Youth?

Systematic reviews and meta-analyses of randomized trials published over the last decade clearly demonstrate that behavioural interventions that target dietary changes and increased physical activity lead to favourable changes in body composition in obese youth [34–41]. Intensive dietary interventions without adjunct exercise therapy elicit a ∼2 % (95 % CI −2.40 to −1.23 %) weight loss defined as weight, BMI, % body fat, waist circumference or skinfold thickness, relative to controls receiving standard dietary recommendations. In a recent position statement from the Academy of Nutrition and Dietetics [37], four dietary strategies for eliciting weight loss in overweight and obese youth were recommended: (1) modified traffic light diet [42], (2) low carbohydrate diet [41, 43, 44], (3) reduced glycaemic load diets [45, 46] and (4) non-diet approach [47, 48, 49••, 50]. Systematic reviews have determined that these strategies are effective in improving body composition in the short term among overweight and obese youth, particularly young children. As all dietary strategies appear to yield similar effects on measures of adiposity, all four options were endorsed.

Fewer studies have examined the effects of exercise alone in improving body composition in obese youth; however, smaller effect sizes were noted in systematic reviews of the available studies [51, 52••, 53]. Exercise trials lasting >16 weeks led to a modest overall −0.4 % (95 % CI −0.7 to −0.1, *P* = 0.006) reduction in percent body fat, −0.2 % (95 % CI: −0.6 to 0.1, *P* = 0.07) reduction in central obesity and a weight loss of −2.7 kg (95 % CI −6.1 kg to 0.8 kg, *P* = 0.07) [54] among overweight and obese youth. A recent trial suggests that among younger obese children, however, supervised, structured endurance exercise offered 4+ times per week yields meaningful reductions in several measures of adiposity, particularly central adiposity, in a dose-response manner [55]. Interestingly, trials comparing the efficacy of adding exercise to a dietary restriction intervention suggest it does not lead to additional weight loss among obese youth [52••], however may yield greater improvements in cardiometabolic health outcomes (reviewed below).

Supervised intensive lifestyle interventions that include both diet and exercise components tend to yield greater weight loss compared with standard weight loss recommendations [53]. Specifically, reductions in BMI (−1.25 kg/m^2^, 95 % CI −2.18 to −0.32) and BMI *z* score (−0.10, 95 % CI −0.18 to −0.02) are significantly greater when both diet and exercise behaviours are targeted in an intensive manner, compared to dietary modification alone. Studies comparing lifestyle interventions to usual care also resulted in significant immediate (−1.30 kg/m^2^, 95 % CI −1.58 to −1.03) and posttreatment effects (−0.92 kg/m^2^, 95 % CI −1.31 to −0.54) on BMI up to 12 months from baseline measures. The effects of these interventions were greater in children (<12 years) than adolescents (13–19 years). Regardless of age, however, the effects were quite variable with reductions as high as −4.3 kg/m^2^ in one study and no effect in several others [53]. The variation in effect size may be related to the intensity of interventions as medium- to high-intensity interventions (>26 h of total contact) yield significantly greater weight loss than low-intensity (<10 h of total contact) interventions (standard mean difference = −1.01, 95 % CI −1.24 to −0.78 vs −0.39, 95 % CI −0.66 to −0.11) [38]. Not all systematic reviews reveal the same effects, as school-based [56, 57], community-based [58] and home-based [59] interventions appear to be ineffective at reducing measures of adiposity in children and adolescents. Cochrane reviews on the prevention [60] and treatment [35] of childhood obesity with modifiable lifestyle approaches suggest that the effects of combined dietary modification and exercise for weight management in obese youth are heterogeneous. Interventions that are more intensive, target younger children (<12 years) and include both exercise and dietary modification are associated with the greatest reductions in measures of adiposity [35, 38, 60]. Taken together, results from these systematic reviews reveal that intensive, structured lifestyle interventions, particularly those that include both exercise and dietary modification, yield modest but meaningful improvements in adiposity in obese children and adolescents. These data suggest that similar strategies may also be beneficial for obese youth living with T2D.

### Is Intensive Lifestyle Management Effective for Cardiometabolic Risk Factor Management?

Among overweight and obese youth, lifestyle interventions confer favourable effects on serum lipoprotein profiles, fitness, insulin sensitivity and systolic blood pressure [52••, 61–64]. A recent systematic review found that lifestyle interventions that elicited significant weight loss in obese youth (see above) yielded small, but significant reductions in low-density lipoprotein cholesterol (−0.30 mmol/L, 95 % CI −0.45 to −0.15), serum triglycerides (−0.15 mmol/L, 95 % CI −0.24 to −0.07), fasting insulin (−55.1 pmol/L, 95 % CI −71.2 to −39.1) and blood pressure (−3.4 mmHg, 95 % CI −5.19 to −1.61) for up to 12 months [53]. Often these improvements are achieved with minimal change in body weight. Importantly, the addition of exercise to a standard dietary restriction approach led to additional reductions in fasting glucose (−2.16 mg/dL; 95 % CI −3.78 to −0.72 mg/dL) and insulin (−2.75 μIU/mL, 95 % CI −4.50 to −1.00) and increased HDL (3.86 mg/dL, 95 % CI 2.70 to 4.63) over a 6-month period [52••]. These effects were observed without any additional weight loss achieved with the addition of exercise to the intervention, suggesting exercise-specific effects are achievable on metabolic health in obese youth.

Interventions aimed at weight loss in obese youth are also effective in reducing systolic blood pressure in the short term [61], regardless of the degree of weight loss. A recent meta-analysis of obesity prevention trials, delivered in large part in school-based settings, revealed an overall reduction in both systolic and diastolic blood pressure of −1.5 mmHg (−0.5 to −2.5 mmHg) [61]. Interventions with the greatest effects on blood pressure were those with a follow-up period <12 months (−2.9 vs −0.3 mmHg), those that included both physical activity and dietary restriction/modification (−2.1 vs +0.11 mmHg vs diet alone) and those delivered in schools (−2.75 vs −0.92 mmHg vs multiple settings) [61]. The effect size for these interventions is rather small from a clinical standpoint; however, these interventions were, for the most part, delivered to healthy weight youth and their impact may be greater in obese youth, particularly those with elevated blood pressure. Similar results have been described in a recent systematic review of trials of exercise training on measures of insulin resistance in youth [62]. Specifically, structured exercise training reduces fasting insulin levels (effect size 0.48 (95 % CI 0.22–0.74, *z* = 3.57, *P* < 0.001) and other measures of insulin resistance (effect size 0.31 (95 % CI 0.06–0.56), *z* = 2.39, *P* < 0.05). The effect of exercise on measures of insulin resistance was greater among youth with an elevated BMI; however, neither the dose, duration nor type of training modified the effect of exercise on insulin resistance. Collectively, results from these systematic reviews suggest that lifestyle-based interventions, particularly those that include exercise, elicit small but significant improvements in several cardiometabolic risk factors, particularly among obese youth.

Arguably, the most successful clinic-based intervention for the management of obesity and cardiometabolic risk factors was developed by Mary Savoye at the Yale Obesity Clinic [48, 49••, 65–67]. Bright Bodies is a 12-month intervention delivered twice weekly for 6 months and weekly thereafter. The intervention includes sessions on goal setting, engineering your environment to make healthier lifestyle choices, mental health, coping and self-regulation among others [48, 49••, 65]. Techniques taught to youth and family members include self-awareness, goal setting, stimulus control, coping skills training (CST), cognitive behaviour strategies and contingency management. Importantly, parents played a key role in the intervention and were taught role modelling of healthy behaviours and coping strategies. The original trial revealed that the intervention was associated with significant reductions in BMI (−3.3 kg/m^2^), body weight (−7.4 kg), body fat (−9.2 kg) and percent body fat (−6.0 %) after 12 months [49••]. The improvement in body composition was associated with significant improvements in total cholesterol (−12.8 mg/dL, 95 % CI −3.8 to −21.9 mg/dL) and fasting insulin (−10.6 μU/mL, 95 % CI −5.7 to −12.1 μU/mL) [49••]. These effects also translated into an increased rate of remission from impaired glucose tolerance in obese youth with dysglycaemia [66, 67]. The positive effects of the intervention remained evident up to 4 years after the intervention ended [48]. The effects of this intensive, clinic-based multidisciplinary model, suggest that under ideal conditions, clinically relevant weight loss and positive metabolic health outcomes are achievable and sustainable in obese youth. As we will discuss below, unfortunately translating these results to youth with T2D prove to be challenging.

In contrast to the wealth of experimental trials of lifestyle therapy for biological outcomes, significantly fewer trials exist examining the effect of lifestyle behaviours on mental health outcomes in obese youth. The Creating Opportunities for Personal Empowerment (COPE) intervention provides promising evidence that the inclusion of educational materials that promote self-efficacy, problem solving, stress management, coping and communication can positively influence both mental and biological health outcomes in overweight adolescents [68]. Compared to standard intervention that promoted simple healthy living messages, the COPE-enhanced programme led to significant short and long-term reductions in adiposity, improvement in social skills and lower substance use in overweight adolescents [68]. A recent systematic review revealed that among children and adolescents, the promotion of structured physical activity, particularly within schools, is an effective approach for reducing systems of depression [69]. The effects were particularly robust among adolescents older than 13 years and those that are overweight or obese. Currently, there is a significant gap in our understanding of the efficacy of the inclusion of positive mental health strategies within healthy living programmes to support positive mental health among obese adolescents. As poor mental health, in the form of depression, anxiety and low quality of life, is a hallmark complication of obese youth, strategies to enhance mental health should be considered key features of behaviour modification approaches for promoting healthy living, particularly among youth with T2D.

### Recommendations for Research

With regard to the prevention of T2D in obese youth, (1) there are no trials of obese youth with impaired glucose tolerance to determine the optimal approach to remission or prevention of incident T2D; (2) there are no trials describing the role of lifestyle therapy on measures of depression, anxiety and coping and (3) few trials have assessed the benefit of adding positive mental health curricula to standard lifestyle interventions on adiposity and metabolic health outcomes.

## Lifestyle Management for Youth with T2D

### Do Changes in Healthy Living Enhance Outcomes in Youth with T2D?

The American Academy of Pediatrics recently released a technical report on the management of T2D in children and adolescents [13] and a series of clinical practice guidelines [70•]. Recommendations for youth with type 2 diabetes include (1) incorporating the Academy of Nutrition and Dietetics’ Pediatric Weight Management Evidence-based Nutrition Practice Guidelines and (2) encouraging that children engage in moderate to vigorous exercise for at least 60 min daily and (3) limiting nonacademic “screen time” to less than 2 h daily [13]. Despite these recommendations, youth with T2D generally achieve significantly less physical activity [17], are generally more sedentary [71] and display lower cardiorespiratory fitness [17, 72] relative to peers without diabetes. Furthermore, youth with T2D display lower diet quality compared to peers with type 1 diabetes [73]. Specifically, they consume twice the amount of sugar-sweetened beverages as peers with type 1 diabetes, less likely to meet recommendations of <10 % of their daily intake of calories from saturated fat, consume fewer micronutrients and report consuming more than they would like to [73]. In light of these poor lifestyle behaviours, strategies to promote healthy lifestyle behaviours should be considered paramount in the treatment of hyperglycaemia and comorbidities in youth with T2D.

In contrast to the wealth of experimental trials of lifestyle-based behaviour modification approaches for normoglycaemic overweight and obese youth, very little experimental evidence exists for the efficacy of lifestyle changes on cardiometabolic health outcomes in youth with type 2 diabetes [13, 32]. Unfortunately, the majority of evidence for the role of lifestyle approaches to clinical management of glycaemia and comorbidities in youth with T2D come from observational studies and retrospective chart reviews [18, 70•, 74–77]. For example, in an effort to understand the effectiveness of lifestyle interventions for glycaemic control in youth with T2D, we performed a retrospective analyses of 275 youth with T2D treated at our centre between 1995 and 2010 [15]. Of the 80 youths that presented with an HbA1c <9.0 % at diagnosis and had a follow-up measurement of HbA1c at 12 months, only 54 % (14 % of the total clinical population) were able to achieve target glycaemic control (HbA1c <7.0 %) and sustain it for 12 months with lifestyle changes alone [15]. A similar chart review of youth treated for T2D in centres in Germany and Austria revealed that very few are treated with lifestyle therapy alone (<20 %) [78] and poor adherence to therapy and withdrawal from care made it difficult to assess the long-term effectiveness of lifestyle therapy alone [78]. A similar trend was observed in youth treated for T2D in the UK, where lifestyle interventions alone were ineffective for glycaemic control [6]. In the absence of therapeutic trials, these clinical observations suggest that intensive lifestyle therapy alone is ineffective for the majority of youth with T2D. This is a theme that is reflected in current clinical practice guidelines [13].

While chart reviews do not support the effectiveness of lifestyle therapy for the treatment of T2D in adolescents, a series of observational studies suggest that various lifestyle behaviours are associated with enhanced cardiometabolic risk profiles [71, 77, 79–81]. For example, youth treated with lifestyle therapy alone tend to display better glycaemic control than those treated with oral hypoglycaemic agents and insulin; however, the cross-sectional nature of the study makes it impossible to know the direction of this association [79]. In analyses restricted to youth with T2D, better diet quality (characterized by achieving adherence to the Dietary Approaches to Stop Hypertension (DASH) recommendations) was associated with a better cardiovascular risk profile [80] and an attenuated rise in systolic blood pressure over 5 years [82]. Similarly, decreased time spent watching television was associated with a significantly attenuated 5-year increase in HbA1c (1.1 vs 2.2 %, *P* < 0.05) and serum lipoprotein levels among youth with T2D [81]. While physical activity are generally lower in youth with T2D compared to peers with type 1 diabetes (4959 ± 3474 vs 7413 ± 3415 steps/day) [83], higher levels of activity are associated with better glycaemic control [77] and self-image [83] in youth with T2D, suggesting that increased activity may enhance self-efficacy among youth with T2D. In summary, (1) a small sample of youth with T2D are able to achieve and maintain healthy living behaviours; (2) the adoption and maintenance of these behaviours are associated with a better cardiometabolic risk profile and (3) physical activity, sedentary time and components of the DASH diet are potential target behaviours for improving health outcomes in youth with T2D. Unfortunately, these behaviours are difficult to achieve and do not translate into better control or remission to normoglycaemia in youth with T2D.

### Experimental Trials of Healthy Living Behaviours in Youth with T2D

To the best of our knowledge, only one therapeutic trial [10••] and one retrospective analysis [84] have examined the efficacy of lifestyle therapy for the management of hyperglycaemia in youth with T2D. The retrospective analysis examined the results of 20 consecutive newly diagnosed children with T2D (14.5 ± 0.4 years) admitted to hospital for treatment with a ketogenic very-low-calorie diet [84]. Adolescents were poorly controlled (HbA1c = 8.8 ± 0.6 %) and obese (BMI = 43.5 ± 1.8 kg/m^2^) prior to being admitted to a clinical research centre to undergo a supervised ketogenic diet. Adolescents were placed a diet of 680–800 cal daily that was high in protein (1.5 g/kg body mass), low in carbohydrates (<30 g daily) and supplemented with micronutrients and vitamins [84]. After 3 days on the very-low-calorie diet, the average blood glucose fell from 8.9 ± 1.1 to 5.5 ± 0.4 mmol/L and nearly all patients discontinued hypoglycaemic medications. The diet resulted in a 12 % reduction in BMI after 6 months, which was maintained for up to 24 months following initiation of the diet. At the end of the diet (average 60 days), HbA1c dropped from 8.8 ± 0.6 % to 7.4 ± 0.6 % and mean arterial pressure fell from 88.6 ± 2.6 to 82.8 ± 1.9 mmHg [84]. The magnitude of change in HbA1c was significantly associated with the time spent on the diet. Changes in BMI and HbA1c were significantly greater than those achieved in a cohort of patients matched for demographic variables that were not placed on the very-low-calorie diet [84]. This study demonstrates that if strict adherence to a hypocaloric diet is maintained, significant improvements in glycaemic control and blood pressure are achievable with lifestyle modifications in youth with T2D. Unfortunately, this approach is not practical in a real-world setting, and these effects have been difficult to replicate clinically.

The only large-scale therapeutic trial to assess the effectiveness of lifestyle therapy for the treatment of glycaemic control in youth with T2D was the Treatment Options for Type 2 Diabetes in Adolescents and Youth (TODAY) trial [10••]. The elements of the intervention and justification are publically available [33]. Behavioural targets included (1) 200–300 min of moderate- to vigorous-intensity exercise weekly, (2) 1200–1500 daily calorie intake and (3) a reduction in baseline body weight by 7–10 %. The diet modification followed the traffic light diet; the physical activity modification relied on a modified traffic light plan with the use of pedometers [33]. Parental support was also a key determinant of the lifestyle intervention that included appropriate praise and reward systems and role modelling of healthy living behaviours [33].

The TODAY trial compared the effectiveness of this intensive lifestyle intervention, combined with daily metformin, to rosiglitazone and metformin and metformin alone on the maintenance of target glycaemic control in 699 youths with T2D that achieved target glycaemic control (HbA1c <7.0 %) prior to randomization [10••]. After 6 months of intensive lifestyle therapy combined with daily metformin, youth experienced a significantly greater reduction in BMI (−0.21 vs +0.35 kg/m^2^, *P* = 0.021), fat mass (−1.04 vs −0.01 %, *P* = 0.006) and absolute fat mass (−0.57 vs +0.69 kg, *P* = 0.029) compared to metformin alone [29]. The greater improvements in body composition were not sustained at the 24 month follow-up visit [29]. Interestingly, while the change in body composition was significantly associated with improvements in insulin sensitivity and HbA1c [29], the additional weight loss associated with intensive lifestyle did not translate into better maintenance of glycaemic control [10••]. Specifically, rates of glycaemic failure (46 vs 51 %) and time to failure (11.8 vs 10.3 months) were similar in the intensive lifestyle group combined with metformin compared to metformin alone [10••].

Intensive lifestyle therapy also did not lead to significant improvements in cardiometabolic risk factors, compared to metformin alone [85]. These data were surprising considering the results of meta-analyses of lifestyle interventions in obese youth presented above. In the absence of adequately powered trials assessing alternate approaches to intensive lifestyle therapy, the results from the TODAY trial suggest that intensive lifestyle therapy is not an effective approach for the management of hyperglycaemia or cardiometabolic risk in youth with T2D. Several factors may explain this failure and are important in the clinical management of youth with T2D.

### Recommendations for Research

With regard to lifestyle management for youth with T2D, (1) there are no therapeutic trials of intensive lifestyle therapy alone for the management of hyperglycaemia and cardiometabolic risk, (2) there are no trials describing the role of lifestyle therapy on mental health outcomes nor their association with clinical outcomes and (3) more in-depth studies of youth that successfully adopt and maintain healthy living behaviours are needed to understand predictors of successful adoption of lifestyle management.

## Why Do Not Lifestyle Interventions Work for Youth with T2D?

### Physiological Factors that Limit the Efficacy of Lifestyle Management in Youth with T2D

There are several key differences between youth and adults with T2D that may explain the lack of effectiveness of intensive lifestyle therapy for achieving significant and sustained reductions in glycaemic control in youth with T2D. First, and most importantly, the pathophysiological and natural history of the disease appears to be markedly different between adolescents and adults [86]. While in adults the onset and progress of hyperglycaemia take years to develop, they both occur more rapidly in youth. Data from the TODAY trial revealed that glucose-stimulated insulin secretion (i.e. beta cell function) declined 20–35 % annually, a rate 3–5-fold faster than reported rates in adults [86, 87]. As glycaemic control is dependent on a coupling of beta cell function and insulin sensitivity [88], an intervention designed to maintain or improve glycaemic control would therefore need to enhance insulin sensitivity at a rate commensurate or greater than the loss of beta cell function. Systematic reviews of intensive lifestyle intervention in obese youth without T2D, suggest that insulin sensitivity may improve ∼20–25 % immediately following the intervention [62], however these improvements are rarely sustainable. To date, the only intervention capable of achieving such a dramatic and sustained improvement in insulin sensitivity is bariatric surgery [89–91], which in most cases, leads to immediate normalization of hyperglycaemia [89–91].

### Mental Health and Social Considerations for Lifestyle Therapy in Youth with T2D

While the unique physiological factors affecting youth with T2D are important in treatment, the psychosocial factors are arguably the most important barrier to lifestyle modification for persons with chronic disease [92]. This is particularly relevant for youth living with chronic diseases, especially if it is compounded by obesity [93], as there is a significant mental health burden associated being obese as a child or adolescent [94]. In a landmark case-control study, obese adolescents reported significantly poorer quality of life and emotional, social and school functioning, compared to healthy weight children [95••]. In fact, impaired quality of life was 5.5-fold higher (95 % CI 3.4–8.7) compared to healthy weight children and no different than children living with cancer (OR 1.3; 95 % CI 0.8–2.3). More recent studies reveal that obese youth, particularly girls, suffer from higher rates of depression and anxiety, relative to healthy weight peers [93]. Several comprehensive reviews have been published over the past 10 years summarizing the burden of depression, anxiety and stress among obese youth [96–98]; however, few studies have turned their focus to obese youth with type 2 diabetes. This is a key gap in the literature as the psychological comorbidities associated with obesity are compounded by the burden of chronic disease self-management. We argue that the psychological comorbidites that cluster in youth with type 2 diabetes are another significant barrier to adopting healthy living behaviours.

The hypothesis was derived from observations made in the clinic in Winnipeg, and from preliminary results from a cohort study of youth with T2D (iCARE) examining the association between mental health and complications [99], as well as results from the TODAY study group [18] and a series of qualitative studies of youth with T2D in our research centre [100, 101]. For example, in a qualitative study that included 28 repeated in-depth interviews and visual arts sessions with eight adolescents with T2D [101], children with T2D revealed two primary themes that guide their self-care behaviours: excessive responsibility and fear. While youth were often perceived as irresponsible by providers and care givers, they reported significant daily responsibilities including caring for younger siblings and family members, who may also be suffering from T2D [101]. A second qualitative grounded theory study of youth, parents and care providers revealed that lack of peer support, mental health issues and bullying were primary barriers to adopting healthy living behaviours [100]. Cultural and socio-ecological differences were also noted as key barriers to implementing healthy living targets within families. Collectively, these data suggest that novel behaviour modification approaches are needed to engage youths and families in adopting healthy living behaviours to support self-management of T2D.

Social determinants of health are cornerstone in the adoption of healthy living behaviours in youth with T2D. For example, youth-onset T2D disproportionately affects youth of ethnic/minority groups and the socially disadvantaged [18, 20•, 26, 102]. Within the TODAY (*n* = 699) cohort, 70 % lived in a single-parent household, 42 % lived in households with an annual income less than $25,000 and 26 % had a parent/guardian with less than a high school education [18, 102]. In our clinic in Winnipeg, 59 % of youth treated for T2D live in a household within the lowest socioeconomic class for the province [20•, 26]. These high rates of social distress combined with the lifelong complex medical regimen associated with T2D may negatively impact the mental health continuum in youth with T2D and, as mentioned above [101], overwhelm youth, making personal well-being a lower immediate priority.

Indeed, rates of depression are common among youth with T2D [24] and threefold higher in youth with type 1 diabetes compared to the general population [103, 104•]. In a population-based sample from the USA, 18 % of youth with T2D had moderate to severe depression, a rate threefold higher than youth with type 1 diabetes [104•]. While depression is an important construct, it is also important to have a better understanding of other key factors involved in mental health including emotional distress (symptoms of depression and anxiety), stress and positive psychological factors and their role in the management of T2D in youth. In fact, ∼20 % of youth with T2D present with a psychiatric disorder or neurodevelopmental/behavioural problems at diagnosis [24]. Moreover, youth with T2D report their quality of life is lower than peers with type 1 diabetes [104•]. The increased burden of poor mental health and low quality of life may explain the failure of lifestyle management, noted by many clinicians and reported in several chart reviews. These findings also reinforce the need for behavioural interventions targeted at social determinants of health and/or resilience-based approaches may be more appropriate for engaging youth and their families in adopting healthy living behaviours that support self-management targets.

## How Do We Promote Healthy Living in Youth with T2D?

### Peer and Social Support

In an effort to overcome limitations of previous interventions, our group has recently tested the effectiveness of peer-led approaches to adopting healthy living behaviours in youth at risk for T2D [105, 106]. Peer-based models for supporting self-management of chronic diseases in adolescents are not novel [107, 108]. Two previous obesity prevention trials demonstrated that peer [109] and social support [110] are more effective that individual weight-loss strategies in youth. As adolescents with T2D often report feeling isolated from peers, marginalized and that support for behaviour change is key to changing lifestyle, we argue that peer-led approaches may be more appropriate for behaviour change that supports lifestyle targets in youth with T2D.

In the two trials performed in our centre, we found that young children receiving curriculum that supported healthy living behaviours from older peers experienced significantly greater reductions in measures of adiposity and improvements in healthy living knowledge [105, 106]. In both trials, peer mentoring was delivered within the school setting using established curriculum grounded in validated theoretical frameworks. In one case, working with youth in a northern isolated Indigenous community [106], the theoretical framework was based in a resiliency centred framework [111, 112]. This approach targets self-efficacy and avoids direct behaviour modification for lifestyle management. We adopted this model in response to previous observations that youth living with and at risk for T2D often require programmes more relevant to their immediate needs. Future therapeutic trials of lifestyle therapy for youth with T2D should consider culturally appropriate approaches that are tailored to the needs to adolescents.

### Social Networks and Social Media

Social media is paramount in the lives of adolescents and significantly influence their behaviour [113, 114]. The American Heart Association recently released a statement regarding the efficacy of social networks in the prevention and management of childhood obesity [115]. There is significant evidence that behaviours related to weight are associated with individuals within social networks [116], in some cases in a dose-response manner [117]. As overweight and obese youth are more likely to be socially isolated [118], the use of social media may be an attractive approach to support behaviour modification, particularly using a peer-based approach.

Systematic reviews of web-based approaches to behaviour modification in youth revealed mixed results [119]. In most cases, internet-based approaches lead to changes in lifestyle behaviours and in some cases reductions in adiposity. The effects of the interventions are often modest; however, these approaches remain in their infancy. Future studies aimed at behaviour modification for lifestyle management in youth with T2D may want to consider these approaches.

### Recommendations for Research

In the context of lifestyle therapy for youth with T2D, there is a dire need for novel approaches to behaviour modification. We recommend that researchers focus their attention on (1) piloting novel approaches to engaging adolescents in programming that appeals to them; (2) considering the use of peer-led approaches whereby older peers, ideally of the same ethnic background, that have successfully adopted lifestyle behaviour work with adolescents to support behaviour change and (3) exploring and piloting social networks to determine if they are an effective tool for engaging youth in behaviour change.

## Conclusions

In conclusion, lifestyle interventions that include exercise and diet elicit modest but potentially meaningful changes in measures of adiposity and cardiometabolic risk in obese children and adolescents. The effectiveness of the intervention is greater with more intense interventions and when delivered at a younger age. While these studies imply that similar effects are possible for obese youth with type 2 diabetes, there is little empirical evidence to support the efficacy of lifestyle interventions for the treatment of youth with type 2 diabetes. The reasons for this are multifactorial. Youth with type 2 diabetes experience more significant and rapid declines in beta cell function and suffer from disproportionately higher levels of mental problems and social adversity. Novel interventions are needed to address these issues, and a larger focus on the poor mental health and quality of life among adolescents with type 2 diabetes needs to be addressed clinically and within future research studies.
